# Apoyo a la lactancia materna en una unidad de cuidados neonatales*

**DOI:** 10.15649/cuidarte.2946

**Published:** 2023-12-20

**Authors:** Sandra Catalina Ochoa Marín, Adrián Arboleda Jurado, Evelyn Manuela García Madrid, Isabel Cristina Arroyave

**Affiliations:** 1 . Universidad de Antioquia. Medellín, Colombia. Email: catalina.ochoa@udea.edu.co Universidad de Antioquia Universidad de Antioquia Medellín Colombia catalina.ochoa@udea.edu.co; 2 . Universidad de Antioquia, Medellín, Colombia. Email: adrian.arboleda@udea.edu.co Universidad de Antioquia Universidad de Antioquia Medellín Colombia adrian.arboleda@udea.edu.co; 3 . Universidad de Antioquia, Medellín, Colombia. Email: manuela.garcia6@udea.edu.co Universidad de Antioquia Universidad de Antioquia Medellín Colombia manuela.garcia6@udea.edu.co; 4 . Hospital San Vicente Fundación Medellín, Medellín, Colombia. Email: isaarroyave@hotmail.com Hospital San Vicente Fundación Medellín Colombia isaarroyave@hotmail.com

**Keywords:** Lactancia Materna, Promoción de la Salud, Enfermería Neonatal, Recién Nacido Prematuro, Unidades de Cuidado Intensivo Neonatal, Breast Feeding, Health Promotion, Neonatal Nursing, Infant, Premature, Intensive Care Units, Neonatal, Aleitamento Materno, Promogáo da Saúde, Enfermagem Neonatal, Recém-Nascido Prematuro, Unidades de Terapia Intensiva Neonatal

## Abstract

**Introducción:**

La lactancia materna (LM) hace parte fundamental del cuidado integral que ofrecen los profesionales de enfermería en la Unidad de Cuidados Neonatales (UCN). Debido a la carga laboral y la delegación de diferentes acciones de cuidado, las dinámicas en estas unidades se centran en la innovación por parte del uso tecnológico, y postergan la humanización del servicio perjudicando el apoyo a la LM.

**Objetivo:**

Diseñar un programa de apoyo a la LM en la UCN.

**Materiales y métodos:**

Investigación cualitativa acción participación. Se realizaron 5 entrevistas semiestructuradas a profesionales de la salud expertos en LM, unos que trabajan en la unidad de la institución en que se realizó el estudio y otros externos, indagando, desde su experiencia y conocimiento, los elementos clave para el diseño de un programa de apoyo a la LM en la UCN.

**Resultados:**

Se exponen en las categorías: a. Cultura en lactancia, comprende: política institucional en lactancia y estandarización del proceso; b. Calidad, comprende: registro y control de actividades y; c. Seguimiento de indicadores y auditoría constante.

**Discusión:**

Se diseñó un programa de LM el cual será aplicado por la institución con el fin de que la LM exclusiva no se vea frustrada, por medio del acompañamiento, la capacitación, la estandarización y la promoción.

**Conclusión:**

El personal de enfermería está interesado en que se estandarice un programa que permita que la información brindada a los pacientes sea veraz y concisa, plantean la educación como pilar fundamental para el desarrollo de un programa exitoso en LM.

## Abstract (en)

**Introduction:**

Breastfeeding (BF) is an essential part of the comprehensive care nurses provide in the Neonatal Care Unit (NCU). Due to the workload and delegation of different care activities, the dynamics in these units focus on innovation using technology and postpone humanization of service, undermining BF support.

**Objective:**

To design a BF support program at NCU.

**Materials and methods:**

Qualitative participatory action research. Seven semi-structured interviews were conducted with health professionals with expertise in BF. Some of them worked in NCU of the health facility where the study was conducted; others were external. Their experience and knowledge were used to identify the key elements for designing a BF support program at the UCN.

**Results:**

They are presented in the following categories: a) breastfeeding culture, including institutional policy on breastfeeding and standardization of the process; b) quality, including registration and control of activities; and c) monitoring of indicators and continuous auditing.

**Discussion:**

A BF program has been designed and it will be implemented by the health facility to prevent the interruption of exclusive BF through support, training, standardization, and promotion.

**Conclusion:**

Nurses are interested in standardizing a program that allows them to provide patients with truthful and concise information, and they view education as a fundamental pillar in the development of a successful BF program.

## Resumo

**Introdujo:**

O aleitamento materno (AM) é parte fundamental da assistencia integral oferecida pelos profissionais de enfermagem na Unidade de Terapia Neonatal (UTIN). Devido a carga de trabalho e a delegagáo de diferentes agóes assistenciais, a dinámica nessas unidades se concentra na inovagáo por meio do uso da tecnologia e adia a humanizagáo do atendimento, em detrimento do apoio ao AM.

**Objetivo:**

Elaborar um programa de apoio ao AM na UTIN.

**Materiais e métodos:**

Pesquisa qualitativa de agáo-participativa. Foram realizadas sete entrevistas semiestruturadas com profissionais de saúde com experiencia em AM, alguns trabalhando na unidade da instituigáo onde o estudo foi realizado e outros de fora, indagando, a partir de sua experiencia e conhecimento, sobre os elementos-chave para a elaboragáo de um programa de apoio ao AM na UTIN.

**Resultados:**

Eles sáo apresentados nas seguintes categorias: a. Cultura da amamentagáo, incluindo: política institucional sobre amamentagáo e padronizagáo do processo; b. Qualidade, incluindo: registro e controle das atividades e; c. Monitoramento de indicadores e auditoria constante.

**Discussao:**

Um programa de AM foi projetado e será implementado pela instituigáo para garantir que o AM exclusivo náo seja frustrado por meio de acompanhamento, treinamento, padronizagáo e promogáo.

**Conclusao:**

A equipe de enfermagem está interessada na padronizagáo de um programa que permita que as informagóes fornecidas aos pacientes sejam verdadeiras e concisas, e considera a educagáo como um pilar fundamental para o desenvolvimento de um programa de AM bem-sucedido.

## Introducción

La LM es fundamental para el cuidado integral que deben ofrecer los profesionales de enfermería en la UCN; proporciona múltiples beneficios a los recién nacidos como fortalecimiento del sistema inmune, prevención del sobrepeso en los niños, aporta al desarrollo cognitivo y promueve el vínculo madre-hijo^1^.

Las enfermeras cumplen un papel relevante en la educación a los cuidadores para el alcance de los conocimientos necesarios y prosperar hacia una adecuada LM^2^; este acompañamiento debe ser individual, planeado y centrado en la familia de manera humanizada, respetuosa y empática^3^.

El tipo de personal que tiene la responsabilidad directa de ayudar a las madres en la sala neonatal con la LM y la alimentación infantil son las enfermeras que trabajan en la unidad^4^, sin embargo, debido a la carga laboral, no se logra operar de manera exitosa los principios de integralidad, equidad, igualdad y calidad que requiere la atención en salud^5^^,^^6^^,^^7^^,^^8^; la rentabilidad financiera y la innovación por parte del uso tecnológico se priorizan por encima de la humanización del servicio, lo que reduce las posibilidades de brindar cuidado de alta calidad^6^^,^^7^^,^^8^. El apoyo a la LM es uno de los principales afectados, lo que lleva a que se delegue o a que se suprima el acompañamiento a los padres^9^, a pesar de que las enfermeras tienen el conocimiento, la habilidad y la experiencia para hacerlo de manera cercana a la familia y según evidencia científica^10^.

El personal de enfermería que labora en la UCN debe ejercer un verdadero rol de liderazgo que promueva la conexión entre enfermera-cuidador-paciente, lo cual “implica un juicio de valor y un proceso dinámico y participativo para identificar y dar prioridad a las necesidades y decidir el plan de cuidado de enfermería”^11^. En consecuencia, la autonomía profesional cobra relevancia en la toma de decisiones, la distribución de los recursos, manejo de conflictos, comunicación y selección de estrategias adecuadas a fin de motivar a las personas que conforman el equipo multidisciplinar a trabajar en pro de la LM^12^.

Algunos estudios demuestran que la promoción de lactancia a través de programas de intervención aumenta el éxito en la alimentación:

Un estudio realizado en una Unidad de Cuidados Intensivos Neonatales (UCIN) de Irán, en el 2013, al organizar dos grupos de bebés prematuros hospitalizados, uno de control en el cual recibieron capacitación y apoyo de rutina sobre la re-lactación, y un grupo de intervención que recibieron capacitación de rutina como del programa de re-lactación educativo y de apoyo que incluye extracción de leche, aumento de los agentes lácteos y cuidado de madre canguro, demostró que existe un aumento significativo del peso de los neonatos cuando se recibe suficiente protección de las enfermeras y del equipo sanitario, además de que la presencia de una enfermera especializada en la UCIN es, por tanto, fundamental para la protección, ofreciendo educación periódica y seguimiento diario^13^.

En China; en el 2019, se implementaron estrategias para la promoción de la LM en prematuros de una UCIN. Establecieron un equipo multidisciplinario experto en LM y en estrategias para el apoyo de esta, guiando la atención integrada en la familia, en la madre canguro y en el banco de leche materna de donantes; se formó un grupo de control y un grupo de intervención. Se demostró que la estrategia de promoción de la LM mejoró la calidad del protocolo ordinario de lactancia. El tiempo hasta el inicio de la alimentación enteral, el tiempo para lograr la lactancia materna completa y el tiempo para lograr la alimentación enteral completa para los bebés prematuros en la UCIN se redujo^14^.En China, en 2021, un estudio demostró que la lactancia entre las madres de RN prematuros en un hospital requiere no solo conocimientos científicos, sino también, un entorno solidario y una práctica centrada en la familia, esto tras implementar un programa que incluyó tres fases: (a) encontrar la mejor evidencia para promover la lactancia materna en la literatura e identificar las brechas entre las mejores prácticas y la práctica actual, (b) implementar estrategias de mejores prácticas, y (c) comparar los resultados previos y posteriores a la intervención. Con base en lo anterior, se implementaron estrategias en la práctica para promover la LM entre las mujeres separadas de sus recién nacidos prematuros^15^.

Objetivo general: Diseñar un programa de apoyo a la LM en la UCN, según personal de salud experto en el tema.

## Materiales y Métodos

Investigación cualitativa acción participación. Se buscó diseñar un programa de apoyo a la LM, a través de la contribución activa de las personas expertas involucradas en el acompañamiento a las madres y sus hijos que se encuentran hospitalizados.

Esta investigación se llevó a cabo en la UCN de un Hospital de la ciudad de Medellín durante un período de 8 meses. El proyecto cumples los aspectos de la Resolución 8430 de 1993 la cual regula la investigación con seres humanos en Colombia. También fue revisado y avalado por el Comité de ética de investigación de La Facultad de Enfermería el 15 de septiembre del 2017 en el acta N° CEI-FE 2017 39. Los miembros del equipo involucrados en el apoyo a la LM en la unidad son enfermeras, auxiliares de enfermería, médicos, nutricionistas, fisioterapeutas, fonoaudiólogos, trabajadores sociales, entre otros. Para la recolección de datos se siguieron diferentes pasos:

1. a. Revisión de políticas y dinámicas en lactancia de la institución: se revisaron protocolos y guías institucionales acerca de la lactancia materna y la estrategia Instituciones Amigas de la Mujer y la Infancia (IAMI), estrategia de la cual el hospital está certificado desde 2006. Además, se realizaron visitas guiadas en la unidad neonatal con una enfermera profesional, en las que se logró observar las dinámicas del servicio en cuanto al proceso de atención y cuidado de los neonatos y sus familias, la infraestructura, la logística y los recursos físicos y humanos que permiten el funcionamiento de la sala de lactancia.
2. b. Entrevistas semiestructuradas (ESE): se contactaron a dos expertas conocidas por los investigadores, una interna a la institución en que se realiza el estudio y otra externa al mismo; se implementó la estrategia de bola de nieve; las expertas se contactaron a través de sus correos electrónicos. Criterios de inclusión: profesionales de salud con estudios complementarios de apoyo a LM que estuvieran debidamente certificados; no se realizó exclusión por tiempo de experiencia como profesional o experiencia en la UCN.

Al realizar la investigación en el marco de la pandemia por COVID-19, se entrevistaron a las expertas a través de la plataforma Google meet, fueron grabadas previo consentimiento informado. Se realizaron 5 entrevistas semiestructuradas, en las cuales se indagó acerca de los componentes y estrategias esenciales para el diseño de un programa de apoyo a la LM en la UCN. En la entrevista número 5 se alcanzó la saturación teórica.

1. c. Análisis: las entrevistas grabadas fueron transcritas, además, se realizó una matriz en la cual se categorizó, codificó y se le dio un memo analítico a una unidad de análisis específica, así se resumieron los hallazgos. Los investigadores se reunieron de manera periódica y plantearon los puntos en concordancia y discordancia de las ESE. Se redactaron los resultados y se construyeron los aspectos necesarios para la realización de un programa de apoyo a la LM en la UCN, el cual se validó con el personal implicado en el acompañamiento en lactancia en la unidad. La información validada fue almacenada en Mendeley Data^16^

## Resultados

Se realizaron 5 ESE, de las cuales 5 fueron mujeres, entre las edades de 35 a 50 años, 4 de ellas enfermeras profesionales que desarrollan roles como asesoras, docentes y jefes de enfermería y 1 nutricionista; las 5 participantes contaban con certificación en asesoría en lactancia, 3 de ellas eran trabajadoras dependientes de instituciones y 3 eran trabajadoras independiente, 3 de ellas eran expertas externas de la institución en la cual se desarrolla la investigación y 2 de ellas eran internas; su experiencia laboral en asesoría en lactancia va desde los 5 a los 16 años y el tiempo de experiencia como profesionales del área de la salud va desde los 13 a los 21 años.

En la revisión de documentos en lactancia con los que cuenta el hospital encontramos: Un protocolo en lactancia no específico para la UCN, que en el momento se encontraba en actualización. Sus temas principales son actividades del personal de asistencia, instrucciones para amamantar al RN, indicaciones para la extracción de LM, extracción manual de leche materna, recomendaciones para la madre y para las enfermeras que van a usar el extractor, conservación y administración de la leche materna, características físico-organolépticas de la leche materna, problemas de la madre y dificultades del niño(a) recién nacido hospitalizado. El personal conoce el protocolo, sin embargo, su aplicabilidad se ve reducida en la UCN. Encontramos una estrategia de apoyo par a par, el cual era ampliamente conocido por los usuarios y trabajaban de manera activa antes de la pandemia, en el momento se encontraba en reactivación. Sus servicios son, atención personalizada en las salas de lactancia, reunión educativa tipo taller cada mes para las familias lactantes, servicio de biblioteca y referenciación a redes de apoyo. El indicador más relevante encontrado fue la “proporción de lactantes que egresan con lactancia materna exclusiva”.

Los resultados se agruparon en las siguientes categorías:

a. Cultura en lactancia: las expertas coincidieron en que se debe constituir y basarse en la creación de una "cultura en lactancia", entendiendo la cultura como bien social conformado por elementos cognitivos, creencias, valores, normas, signos y símbolos que construyen las acciones y las actitudes de un grupo social^17^, como se observa en los siguientes testimonios:

*“Esa importancia radica en un cambio para la sociedad, la lactancia no solo es alimento, es un apego seguro, confianza, equidad en salud, es cultura” [ESE 02]. “Cuando se promueve la lactancia exclusiva y continua se va creando una cultura, si una mamá ve lactando a otra, casi que por reflejo, le va a bajar leche, se convierte en una cultura de “si ella puede yo puedo”” [ESE 01].*

Las expertas recomiendan contemplar las siguientes subcategorías con el objetivo de construir cultura en lactancia en la UCN y asegurar el éxito y la replicabilidad del programa:

a.1.Política institucional de promoción de la lactancia: coincidieron en que proteger la lactancia materna es una responsabilidad colectiva, es necesario que cada eslabón de las instituciones coopere y unan fuerzas. Aseguran que desde la administración se debe movilizar al personal y crear alianzas que brinden los recursos necesarios para el bienestar y la protección del derecho a lactar. Esto se evidencia en el siguiente testimonio:

*“Es importante tener el apoyo de las partes directivas de una institución, que estén comprometidos y que le ayuden a las personas que van a encaminar este programa. Los hospitales que tienen unidades neonatales deben garantizar que la madre pueda estar en contacto con el bebé, que tenga acceso a la extracción y suministro de su leche a su bebé, educación sobre lactancia, contacto directo con él, que lo canguree, que lo pueda pegar al pecho según las condiciones de salud del neonato” [ESE 03].*

Sin embargo, según la experiencia, las expertas concuerdan en que convencer a los directivos de movilizar recursos y personal, tanto de planta como en formación, es una tarea difícil. Se han encontrado múltiples barreras debido a que no se ha logrado demostrar el costo - beneficio del apoyo a la LM debido al subregistro de las actividades de acompañamiento a las madres en el amamantamiento, lo que lleva a indicadores insuficientes que no permiten el análisis riguroso de la situación en la unidad. Una experta externa comenta:

*“Si se evidencia ante los directivos que, facilitando una sala de extracción o una sala de lactancia dentro de la misma institución, recurso humano que acompañe a esas mamás para la extracción, la conservación y el suministro de la misma leche a sus recién nacidos, se reduce el tiempo de estancia y costos, seguramente van a tener sus motivos para cambiar sus políticas [ESE 05].*

a.2.Hablar el mismo idioma a través de la capacitación permanente del equipo de salud: estandarización del proceso, atención multidisciplinar y capacitación de los diferentes actores: las participantes externas e internas enfatizan en la importancia de la estandarización del proceso y de conceptos con el fin de llegar a una norma común de atención en lactancia en la UCN, además, de la creación de un equipo multidisciplinar capaz de establecer metas de cuidado que satisfagan las diferentes necesidades de apoyo a las madres que practican la lactancia. Las expertas internas comentan la necesidad de que el programa sea lo más detallado posible en los pasos del proceso de atención en lactancia, ya que, por condiciones institucionales, el personal es limitado y debe rotar por diferentes servicios de manera constante, lo que limita la captación oportuna de las madres y la constancia que requiere el programa para ser exitoso y eficaz. Al respecto se cuenta con los siguientes testimonios:

*“Para mí es completamente necesario y pertinente que todo el personal, y no solo me refiero a enfermería, sino a todo el equipo de salud, que ojalá estén hablando el mismo idioma relacionado con lo que es ese apoyo a la lactancia; que todos apunten a tener una meta común con las pacientes y los RN que tienen allí” [ESE 01].*

*“La idea que yo siempre he tenido es de estandarizar el programa de manera que lo puede hacer la auxiliar o el estudiante, pero sea el mismo programa para todo el mundo, estandarizado institucionalmente para eso. Me preocupa que llegue una persona que le dé un tipo de información diferente a la mamá que complique el asunto, esa parte de la estandarización del proceso y de los conceptos me parece que es importante” [ESE 04].*

El equipo multidisciplinar debe realizar una valoración exhaustiva, individualizada y holística con el objetivo de planear intervenciones justificadas, teniendo en cuenta los intereses y aspiraciones de la madre, además del contexto en el que vive la triada familia-madre-hijo, ya que, las expertas coinciden en que las condiciones sociodemográficas y psicológicas influyen en el apego y el progreso de la alimentación a través de la leche humana. Ellas afirman:

*“Una de las informaciones que se debe dar desde que el neonato ingresa a la unidad son las opciones de lactancia materna dependiendo de lo que quiera la madre y las necesidades que tengan, que haya una persona encargada todos los días de captar a las madres” [ESE 01]. “También es importante tener en cuenta que en el hospital hay muchas mamás que tienen contraindicaciones temporales o definitivas en lactancia, con ellas también se debe trabajar la lactancia ya sea para la re-lactación o el inicio de la lactancia o las opciones en los casos definitivos y como terminar la lactancia” [ESE 03].*

En un servicio de cuidados neonatales se realiza la atención del RN y a su madre con situaciones de compromiso vital, que precisa de medios y cuidados especiales de forma continuada; cuando el equipo no se encuentra sensibilizado sobre las intervenciones específicas para cada situación de la diada madre-hijo y de la familia en general, la meta de alimentación exclusiva a través de la leche humana se vuelve inalcanzable; según las expertas, el personal, debe ser capaz de brindar una atención basada en la evidencia y desarrollar capacidades y aptitudes que les permita responder a las cosmovisiones de la lactancia. Es crucial que exista una motivación genuina y responsable con el cuidado, ya que las expertas aseguran que la adherencia por parte de las madres se da gracias a la capacidad del profesional en transmitir sus conocimientos a través de la creación de un ambiente de confianza y de seguridad. La fuerza de brindar un entorno favorecedor a las personas lactantes radica en que amamantar se convierte en parte de sus vidas diarias, de sus rutinas; el sentirse apoyadas y escuchadas por su contexto social hará de la lactancia un proceso seguro, amoroso y reconfortante:

*“Las personas que trabajen en este programa deben tener ciertas aptitudes para saber llegarle a las madres y poder resolverles todas las dudas de manera integral, yo creo que la consejería es algo muy importante, a parte de los conocimientos que las personas de este programa deben tener en general sobre LM”. “Que el personal esté capacitado y que tenga una vocación y les guste enseñar lactancia” [ESE 02].*

El cuidado en LM es uno de los más humanizantes y centrados en la familia que veremos en los servicios de cuidado neonatal, así que, también se debe contar con la formación y la motivación de la familia y de las madres; un programa que no tenga en cuenta los conocimientos y aptitudes de los cuidadores difícilmente encontrará el camino al éxito. Las expertas aseguran que la educación a las madres debe ser una conversación bidireccional, por lo que se debe evitar brindar información descontextualizada, que no responda a la situación específica de esa madre y del neonato. Ellas coinciden en que debe construirse un proceso de aprendizaje y de empoderamiento de las mujeres que deciden vivir la lactancia, donde los mitos y las barreras no encuentren espacios para ser. Una de las expertas comenta la siguiente estrategia que se podría extrapolar a la lactancia en la UCN:

*"En el hospital no hay un programa de educación para una mamá, para explicarle, por ejemplo, la importancia de la LM. Hay instituciones que tienen cursos de lactancia para las mamás que están en cuidados intensivos y las gradúan, y eso es importante y que sea a cargo de la profesional de enfermería, que sea una persona experta en el tema, así como en otras instituciones hay cursos de reanimación neonatal y signos de alarma para padres, también debería haber uno de lactancia, eso para mí me parece que es de las estrategias más innovadoras, uno realmente con el conocimiento y la educación hace muchísimo” [ESE 05].*

b. Calidad: A lo largo de la investigación las expertas refieren que el control de la calidad debe ir acompañado de un adecuado registro de actividades, el cual permite dar un seguimiento claro y veraz por medio de los indicadores de que el programa está siendo aplicado de manera idónea en la unidad; garantizando que se esté llevando a cabo correctamente.

b.1. Registro y control de actividades: Las expertas coincidieron en que se debe dar un seguimiento a las acciones de los diferentes protagonistas de la atención, por medio del registro de las actividades de manera imprescindible como parte de la asistencia en lactancia. A través de la historia clínica (HC) y las notas de enfermería se puede evidenciar la calidad asistencial, administrativa y el cumplimiento con los estándares de cuidado nacionales e internacionales, además, facilita la comunicación multidisciplinar y brinda autonomía y liderazgo al equipo enfermero en las actividades que realiza:

*“Con solo observar una HC o una nota de enfermería en el último mes usted puede hacer seguimiento si se hizo valoración sobre la lactancia, o puedo entrevistar una mamá y mirar si se incluyó la valoración de lactancia, cómo estuvo su APGAR, por qué no se pegó al pecho inmediatamente, si hubo calostro, qué características anatómicas tuvo el RN y tiene la madre y si favorece o no ese agarre” [ESE 04].*

El registro de la nota de enfermería en lactancia permitirá hacer un seguimiento continuo y constante en el cual se podrá encontrar la información brindada y los hallazgos de importancia encontrados durante la valoración de manera veraz y concisa, esto con el fin de garantizar una continuidad en la atención por parte de todo el equipo multidisciplinario, respetando así las convicciones de la madre y garantizando continuidad en los procesos.

*“Nosotros acá tenemos una nota de protocolo la cual debemos incentivar su uso, es una nota de lactancia donde decimos, qué se habla con la mamá, cómo es el banco de leche, las posturas, como las cosas básicas, yo se la pase a todas las auxiliares del equipo entonces todas tienen la misma nota, está en toda la UCI, es una nota pública en el grupo IAMI, todo el mundo sabe que hay una nota de lactancia materna [ESE 05].*

b.2. Gestión de la atención en lactancia: las expertas a lo largo de la investigación proponen indicadores que deberían ser tomados en cuenta a la hora de la elaboración de un programa en lactancia materna exitoso, pues estos desde diferentes puntos de vista dan frente a la solución de las necesidades que se presentan en LM, desde la institución, la unidad y el personal.

*“Es importante que desde las directivas haya una voluntad política, pero también, desde enfermería que haya un jalonamiento en cuanto lo que es la gestión del servicio, y la gestión no es saber cuántos neonatos entran y salen cada mes, la gestión del servicio es mirar esos indicadores e implementar unos procesos de cuidado y unos planes de mejoramiento según esos indicadores, como bajar la estancia hospitalaria. Que digan “las complicaciones más frecuentes de la lactancia, o las necesidades ha sido está en el recorrido del año, qué estamos haciendo para mejorar esas necesidades, ah que hicimos dos charlas al año, ¿será que si es suficiente?, ¿estamos midiendo si esas mamás que les dan de alta a sus hijos si están cumpliendo con la LM o qué está sucediendo?”. [ESE 01]*

De la mano de las recomendaciones de las expertas para esta investigación, la institución, debe verificar la ejecución y el desempeño de este. Esto se realiza a través de la auditoría; que a su vez está directamente ligada a los indicadores; este es el mecanismo para la evaluación constante respecto a lo que se espera del programa y sus metas de cumplimiento, tanto del personal como de los usuarios.

Cuando este proceso no es riguroso, se corre el riesgo de que los programas no se sostengan en el tiempo, no se da un cumplimiento de los estándares y de las normas, impactando negativamente en la calidad de la atención.

*"Aquí se hace un seguimiento a los 15 días, de cómo va la lactancia materna y mirar en primer lugar, la madre salió con qué tipo de alimentación, si es mixta o exclusiva, sí se tuvo lactancia en la primera hora de vida y el seguimiento posterior, donde ellas refieren si siguen con lactancia, si es exclusiva, si ya es mixta, entonces con eso se dan los indicadores que lleva el hospital" [ESE 05].*

## Discusión

El apoyo a la lactancia materna en la UCN pasa a un segundo plano y se frustra fácilmente por la limitada educación y sensibilización de los profesionales en lactancia materna y la falta de estandarización del proceso de atención. Un “Estudio de estrategias de promoción de la lactancia materna en prematuros en la unidad de cuidados intensivos neonatales” realizado en 2019 en China plantea que la promoción de la misma genera un efecto positivo en los servicios de salud^14^. Al igual que los estudios realizados al interior del país, demuestran que una asesoría por parte de los profesionales mejora notablemente las tasas de lactancia materna exclusiva y eliminan barreras a la hora de lactar^18^.

Un estudio realizado en 2020, titulado “Knowledge and attitude of health staff towards breastfeeding in NICU setting: ¿are we there yet? An Italian survey”, afirma que “la admisión de prematuros en una unidad de cuidados intensivos neonatales (UCN) y las condiciones clínicas del recién nacido se han descrito como barreras importantes, lo que lleva a tasas más bajas de inicio y duración de la lactancia materna”^19^, sin embargo aquí es donde el rol del equipo multidisciplinario debe poner su conocimiento en función de que la lactancia materna no se vea frustrada y por medio del acompañamiento, la capacitación, la estandarización y la promoción se pueda hacer un proceso satisfactorio que beneficie el binomio madre e hijo.

La lactancia, nos lleva a tocar temas como el apego seguro, la confianza, la nutrición, sistemas inmunológicos fuertes y más desarrollados y también beneficios económicos para la familia y las instituciones prestadoras de servicio, como se afirma en “análisis de impacto normativo en lactancia materna” del Ministerio de Salud y Protección Social, “ El incremento de las tasas de lactancia materna exclusiva podría ayudar a impulsar el progreso en otros objetivos globales de nutrición como: disminución del retraso en el crecimiento, anemia en mujeres en edad reproductiva, bajo peso al nacer y sobrepeso infantil, siendo la lactancia, una de las herramientas más poderosas que tienen los formuladores de política a su disposición, para mejorar la salud y economía de sus poblaciones”^20^.

Por último extender esta invitación a las instituciones formadoras de personal en salud de toda índole, para así tener profesionales conscientes, sensibles y capacitados respecto al tema, “Los técnicos y los profesionales de la salud son las personas que deben estar capacitadas y fortalecidas en los temas de lactancia materna para poder orientar a las mujeres y las familias, por lo cual son muy importantes los procesos de formación y actualización que estos reciban de la academia”^21^; los programas académicos de las instituciones no contemplan el desarrollo de temas relacionados con lactancia materna, esta profundización es necesaria para que el personal de salud tenga elementos técnicos y prácticos para brindar un acompañamiento integral.

## Conclusiones

El personal aborda la lactancia materna desde la responsabilidad, el compromiso y la entrega para brindar un cuidado integral al neonato y sus familias, sorteando las barreras con calidad, creatividad y recursividad. Las participantes coinciden que el apoyo a la LM debe incluir un equipo multidisciplinario con una estrategia de comunicación efectiva que direccione hacia unas metas comunes y hacia la estandarización del proceso desde el primer contacto con el neonato y su familia hasta el alta hospitalaria. Los resultados de esta investigación fueron de gran utilidad para la creación de un programa en lactancia materna en formato de cartilla implementada en la atención de la UCN de la institución en la cual se realizó el proyecto.
